# Relevance of chronic stress and the two faces of microglia in Parkinson’s disease

**DOI:** 10.3389/fncel.2015.00312

**Published:** 2015-08-14

**Authors:** Antonio J. Herrera, Ana M. Espinosa-Oliva, Alejandro Carrillo-Jiménez, María J. Oliva-Martín, Juan García-Revilla, Alberto García-Quintanilla, Rocío M. de Pablos, José L. Venero

**Affiliations:** Departamento de Bioquímica y Biología Molecular, Facultad de Farmacia and Instituto de Biomedicina de Sevilla (IBiS), Hospital Universitario Virgen del Rocío/CSIC/Universidad de SevillaSevilla, Spain

**Keywords:** corticosterone, glucocorticoids, microglia, neuroinflammation, neurodegeneration, stress, Parkinson’s disease

## Abstract

This review is aimed to highlight the importance of stress and glucocorticoids (GCs) in modulating the inflammatory response of brain microglia and hence its potential involvement in Parkinson’s disease (PD). The role of inflammation in PD has been reviewed extensively in the literature and it is supposed to play a key role in the course of the disease. Historically, GCs have been strongly associated as anti-inflammatory hormones. However, accumulating evidence from the peripheral and central nervous system have clearly revealed that, under specific conditions, GCs may promote brain inflammation including pro-inflammatory activation of microglia. We have summarized relevant data linking PD, neuroinflamamation and chronic stress. The timing and duration of stress response may be critical for delineating an immune response in the brain thus probably explain the dual role of GCs and/or chronic stress in different animal models of PD.

## Role of Inflammation in Parkinson’s Disease

Parkinson’s disease (PD) is a neurodegenerative disorder second only to Alzheimer’s disease (AD) in prevalence. It is characterized by the loss of the dopaminergic neurons in the substantia nigra (SN) and the accumulation of α-synuclein (α-syn) and other proteins in intracellular proteinaceous aggregates called Lewy bodies (LB). Familiar forms of PD, usually appearing under the age of 40 and accounting for no more than 5% of cases, are due to mutations in a reduced number of genes including *SNCA, PINK1, PARKIN* and *LRRK2* among few others. Idiopathic forms, usually affecting people from 65 years old, have an obscure etiology; mitochondrial dysfunction, toxins, oxidative stress, infections, decrease of trophic factors, impairment of the ubiquitine-proteosome system, metabolic alterations, inflammation and the accumulative effect of a number of susceptibility genes have been proposed to explain the initiation and development of this form, which accounts for 95% of cases.

### Neuroinflammation

Neuroinflammation seems to be an underlying process in many cases of PD. In McGeer et al. (1988) reported the presence of reactive microglia and inflammatory macrophages as well as proinflammatory cytokines in SN postmorten samples from PD patients. Considering the brain was believed to have immune privilege,these inflammatory signs were thought to be a response from the microglial system to neuronal death. The brain is no longer considered to be immunoprivileged; in fact, infiltration of lymphocytes into the brain parenchyma of PD patients has been demonstrated (Brochard et al., 2009); the role of T lymphocytes in PD will be reviewed in “Chronic Stress and Parkinson’s Disease in Humans” Section).

**Figure 1:**
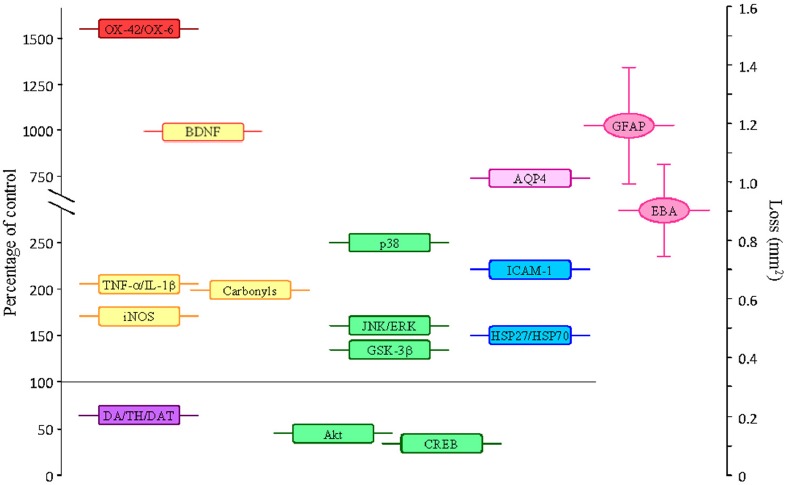
**Average values of some parameters measured in the SN (as percentage of controls) after the single intranigral injection of 2 μg of LPS.** Parameters that increase: OX-42/OX-6, density of activated microglial cells; amounts of the proinflammatory cytokines TNF-α and IL-1β; the inducible nitric oxide synthase (iNOS) enzyme; the amount of carbonyl groups (oxidized proteins); the expression of BDNF (this neurotrophin is associated to cell survival, but it can have a damaging role under the oxidative conditions induced by LPS); the phosphorylated (active) forms of the MAP kinases p38, JNK, ERK and GSK-3β (associated with promotion of apoptosis); the expression of AQP4; the adhesion molecule ICAM-1; the heat shock proteins (HSP)-27 and 70. Parameters that decrease: DA/TH/DAT, dopamine content, neurons expressing tyrosine hydroxylase and dopamine transporter; the phosphorylated forms of the MAP kinase Akt and the transcription factor CREB (cell surviving signals). Alterations on the expression of GFAP and the endothelial barrier antigen (EBA), as area lacking expression (in mm^2^), are also shown. Loss of expression of GFAP and EBA is associated to BBB damage.

It is now thought that neuroinflammation could be a triggering mechanism of neuronal death. Inflammatory animal models based on the injection of proinflammatory compounds as LPS, thrombin or tissue plasminogen activator within the SN have shown that the induction of an inflammatory process can induce the death of dopaminergic neurons (Castaño et al., 1998, 2002; Herrera et al., 2000; Kim et al., 2000; Carreño-Müller et al., 2003; de Pablos et al., 2005, 2006; Tomás-Camardiel et al., 2005; Hernández-Romero et al., 2008; Villarán et al., 2009; Argüelles et al., 2010). Evidence supporting the inflammatory hypothesis of neurodegeneration also comes from studies showing the expression of a bunch of inflammatory markers within the brain including specific proteins, pro-inflammatory cytokines and markers of active glial cells (for a schematic review of the effects of LPS on neurons and glial cells found by our group, see Figure 1). An altered expression of immune signaling-related transcripts have been described in early stages of PD in a study of microarray analysis of nucleated blood cells (Soreq et al., 2008). Epidemiological studies evidence the protective effect of several nonsteroidal anti-inflammatory drugs, whereas genetic studies show that polymorphisms in some pro-inflammatory cytokines may influence the risk of developing PD (Klegeris et al., 2007). Some studies have shown that classical steroid anti-inflammatory drugs, such as dexamethasone (Castaño et al., 2002), as well as drugs used for quite different goals, such as minocycline (Tomás-Camardiel et al., 2004) and simvastatin (Hernández-Romero et al., 2008), are able to reduce the inflammatory process and neuronal death induced by LPS. Thus, it seems that the pro-inflammatory hypothesis is not merely possible but likely. The question here is how such an inflammatory process is initiated within the brain and endlessly self-sustained.

Not all brain structures exhibit a similar sensitivity to pro-inflammatory compounds; whereas the SN seems to be very sensitive, the hippocampus appears to be resistant to LPS (Espinosa-Oliva et al., 2011). This could be in part related to differences in the number of microglial cells between brain structures (Lawson et al., 1990). However, an inflammatory process induced for LPS can be developed in the hippocampus of stressed animals (Espinosa-Oliva et al., 2011), suggesting that the inflammatory responses can be modulated. Unfortunately, the factors triggering this brain inflammatory response are yet not known.

Dopaminergic neurons seem to be especially sensitive to a number of factors that can induce cell damage and eventually cell death. Dying neurons release substances that are recognized by glial cells, activating them. Amongst the substances released by damaged/dying dopaminergic neurons, this review will focus on three of them: dopamine (DA), neuromelanin (NM) and α-syn.

### Dopamine

DA is the neurotransmitter that defines the dopaminergic phenotype. It is synthesized from tyrosine in a two-reaction process catalyzed by the enzymes tyrosine hydroxylase (TH, the key enzyme in the synthetic pathway of DA) and aromatic amino acid decarboxylase (AADC), which transform tyrosine in L-3,4-dihydroxyphenylalanine (L-DOPA) and then L-DOPA into DA, respectively. The loss of DA is the hallmark of PD and explains most of the motor alterations of this disease. Thus, replenishing DA levels is a major therapeutic target in PD. This is achieved by the combined administration of L-DOPA, the precursor of DA, and an inhibitor of peripheral AADC; unfortunately, L-DOPA cannot be freely administered because many patients develop L-DOPA-induced dyskinesias. After its release in synapses and signaling on its receptors, DA can be taken up by glial cells and also by the presynaptic neurons through the specific DA transporter. Uptaken DA can be revesiculated in neurons by the vesicular monoamine transporter 2, but part of it can remain free in the cytoplasm, where it is metabolized by the monoamine oxidase enzyme to yield 3,4-dihydroxyphenylacetic acid. Free cytoplasmic DA can also autooxidize, forming DA quinones and semiquinones able to react with cysteines residues to form adducts that impair the function of proteins; for example, DJ-1, a redox sensor protein involved in recessive forms of familial PD, is covalently modified by DA and highly reactive DA quinones thus impairing DJ-1 function (Girotto et al., 2012). High cytosolic concentrations of free DA can lead to oxidative stress as well as to its interaction with α-syn, both of them initiating the neurodegenerative process (Mosharov et al., 2006). Strikingly, DA seems to play a role in the inflammatory response induced by the intranigral injection of LPS, since experimental depletion of DA caused by the inhibition of TH by α-methyl-p-tyrosine (α-MPT) prevents glial activation and loss of dopaminergic neurons (de Pablos et al., 2005). Bypassing TH inhibition (using the combination of L-DOPA plus an inhibitor of the peripheral AADC enzyme, just the composition of several anti-parkinsonian therapies as SINEMED® or MADOPAR®, for example) reverses the protective effect induced by α-MPT. Furthermore, depletion of DA content by α-MPT is able to reduce the infiltration of peripheral macrophages as well as the increase of microglial cells induced by the injection of 6-hydroxydopamine (6-OHDA) within the SN (de Pablos et al., 2014). Thus, DA could have a relevant role in sustaining inflammation and lymphocyte recruitment within the brain; in fact, DA could be involved in the degeneration of dopaminergic neurons induced by inflammatory processes.

DA, however, may play a beneficial role controlling systemic inflammation. In a recent article, Yan et al. (2015) show that DA inhibits the activation of the NLRP3 inflammasome through D1 receptor (DRD1) signaling. DRD1 activation stimulates adenylate cyclase activity; the resulting cAMP binds to NLRP3, promoting its ubiquitination by the E3 ubiquitin ligase MARCH7. Degradation of the ubiquitinated NLRP3 prevents the inflammasome-dependent inflammation. Acting on DRD1, DA is even able to prevent systemic inflammation induced by LPS, proving that DA has an important role as a messenger in the periphery. Consequently, these authors point out to DRD1 as a potential target for the treatment of inflammatory processes driven by NLRP3.

### Neuromelanin

NM is a dark, complex endogenous polymer derived from DA that stains dopaminergic neurons and give the name to the SN; loss of pigmentation is evident in the SN of PD patients due to the loss of dopaminergic neurons. Free melanin was already observed in the SN of postmorten samples from PD patients studied for McGeer et al. (1988). Several studies have shown that NM is able to induce microglial activation, initiating neuroinflammation and neurodegeneration of dopaminergic neurons (Wilms et al., 2003; Zecca et al., 2008; Karlsson and Lindquist, 2013; Nie et al., 2013). Viceconte et al. (2015) has shown that synthetic NM is able to activate microglial cells, both *in vitro* and *in vivo*, through a mechanism mediated by caspase-8 and caspase-3/7. They found *in vivo* upregulation of the M1 marker CD16/32 induced by NM, as well as chemotactic response to NM for the microglial-derived cell line BV2, accompanied by features of microglial activation as morphological changes, increase of oxidative stress and induction of pattern recognition receptors as TLR2, NOD2 and CD14. Thus, release of NM from damaged/dying dopaminergic neurons can trigger a self-induced cycle of neuron damage, release of NM and neuroinflammation with dire consequences for these neurons.

### Alpha-Synuclein

As it was mentioned above, some cases of familiar PD are due to mutations in a number of genes related with mitochondrial function, so as neurons lose their capacity to produce the required amount of energy and thus die, activating and attracting glial cells. In other cases, polymorphisms in inflammatory genes seems to be associated with the disease, including pro-inflammatory cytokines such as TNF-α and IL-1β and their receptors (Krüger et al., 2000; McGeer et al., 2002; Schulte et al., 2002; Wahner et al., 2007; Wu et al., 2007; Nie et al., 2013), as well as TREM2 (Rayaprolu et al., 2013). An increasing number of GWAS studies have provided possible genetic associations with the disease, as that found for HLA for example (Hamza et al., 2010), supporting the role of the immune system in the development of PD.

A great proportion of these hereditary forms of PD are related to α-syn, a presynaptic protein involved in vesicle transport among other functions not fully known (Maroteaux et al., 1988). α-syn aggregation seems to be an important event in the development of PD (Marques and Outeiro, 2012; Kalia et al., 2013). Several point mutations in the *SNCA* gene are directly related to familial forms of PD (Polymeropoulos et al., 1997; Krüger et al., 1998; Zarranz et al., 2004; Lesage et al., 2013; Proukakis et al., 2013). Over-expression of wild type α-syn due to duplication or even triplication of the gene is also related with PD, suggesting that an excessive amount of the normal protein is also involved in damaging processes (Singleton et al., 2003; Ross et al., 2008).

As mentioned above, interactions between α-syn and DA might play an important role in the death of dopaminergic neurons: oligomeric forms of α-syn can produce leakage of DA from presynaptic vesicles into the cytosol (Volles et al., 2001), and high cytosolic concentrations of DA may in turn promote the accumulation of α-syn protofibrils (Conway et al., 2001). Wild-type α-syn can be modified by DA, resulting in the inhibition of chaperone-mediated autophagy (CMA), which impairs degradation of α-syn and other substrates of CMA (Martinez-Vicente et al., 2008). Furthermore, neuron-released α-syn fibrils that have been phagocytized by microglia can induce the release of IL-1β through the activation of the NLRP3 inflammasome (Codolo et al., 2013). It is well known that IL-1β is one of the main and most abundant pro-inflammatory cytokines affecting inflammatory processes (Dinarello, 2010), having harmful effects on dopaminergic neurons (Ferrari et al., 2006; Koprich et al., 2008). The role of IL-1β on inflammation and dopaminergic neurodegeneration will be discussed later. All these evidence suggest that interactions between α-syn and DA might trigger a self-sustained loop leading to neuronal death.

Braak et al. (2003) postulated the hypothesis that pathology of PD in the brain could start in structures such as the olfactory nucleus and the dorsal motor nuclei, progressing from them to the midbrain and cortex by the spreading of α-syn seeds. The possible mechanism for cell-to-cell transmission of pathogenic proteins in neurodegenerative diseases has been beautifully revised by Guo and Lee (2014). In brief, naked-protein oligomers seeds can be released from neurons, reaching other neurons directly through the plasma membrane or entering by endocytosis. Seeds can also be released within exosomes that fuse with the receiving cell membrane. Intracellular transfer by nanotubes connecting cells is also hypothesized. It is now clear that α-syn can be found outside cells (for a review, see Guo and Lee, 2014), and several studies have shown that α-syn activates microglia into a pro-inflammatory state (for a very interesting and deep review on microglial activation by α-syn, see Sanchez-Guajardo et al., 2015). Thus, α-syn might be an answer to the question stated above: an inflammatory process can be initiated within the brain and endlessly self-sustained upon activation of microglia by extracellular α-syn. However, some questions regarding neuroinflammation and α-syn are still under debate, as whether neuroinflammation is causing α-syn misfolding and aggregation, or the role of neuroinflammation favoring or delaying the cell-to-cell transfer of α-syn; namely, if α-syn aggregation is a cause of neuroinflammation or just a consequence of it (Lema Tomé et al., 2013). In any case, neuroinflammation and α-syn aggregation form a positive feedback loop that increases protein aggregation and neuronal damage in PD. Strikingly, the release of α-syn from neurons, with its consequent arrival to other neurons, can be initiated outside the brain.

Given the significant role of inflammation in PD pathogenesis, and considering the influence of stress/glucocorticoids (GCs) in microglia polarization, we will next discuss most relevant aspects of stress and microglia activation as well as their likely influence on PD.

## Stress

Organisms, both simple and complex, have developed mechanisms to deal with the different challenges that occur during their lifetime. Stress is a condition of human experience and also an important factor in the onset of several diseases, including cardiovascular, metabolic and neuropsychiatric diseases (Renard et al., 2005; Musazzi et al., 2010). Stress is a term commonly used to describe internal states or feelings and, as a consequence, the interpretation of its meaning is often subjective, making it difficult to assess how it impacts upon our body (Miller and O’callaghan, 2005).

The term stress would be difficult to understand without the concepts developed by the physiologists Claude Bernard and Walter Cannon. Whilst the former described the concept of internal environment (Millieu Interieur), the latter introduced the term homeostasis to define the combination of physiological mechanisms that together maintain the stability of the internal environment. Homeostasis is achieved via different systems which cooperatively counteract internal or external factors that tend to modify it. Based on these concepts, Selye (1946) coined the term *stress* as a “general adaptation syndrome”. Of note, the cortico-adrenal activation is the most important physiological aspect of stress. Selye would futher introduce the term *stressor* as the stimulus that causes the syndrome, narrowing the definition of stress as the body’s response to such stimulus. However, although numerous scientific articles on stress have been published in recent decades, the definition of stress remains complex, controversial and somehow ambiguous.

Under stressful situations, humans mobilize physiological resources to respond to these situations in the so-called stress response (Van de Kar and Blair, 1999). Different variables influence the individual response to these threats. Among these stand stressors (depending on the type, severity or repetition of events), individual factors (such as vulnerability, emotional stability and coping styles) and environmental variables. The interaction of these variables can lead to various stress responses from the physiological point of view, in which brain (information processing), autonomic (innate system) and neuroendocrine (adaptative system) activation are involved. Although they are individual systems, in practice their functions overlap at some point. All these responses constantly feedback the body to increase, maintain or decrease the stress response.

### Systems Activated by Stress

When an organism is stressed, two different systems are activated: the Sympathetic-adrenal-medullary system (SMA) and the Hypothalamic-pituitary-adrenal system (HPA). Initially, a threatening stimulus excites a receptor leading this information to the brain in the form of nerve impulses. The sensory information is then projected on the associative thalamic nuclei from where it continues its journey to the cerebral cortex (Van de Kar and Blair, 1999), which is responsible for modulating the sensory processing and identify if there is a threatening stimulus or not. The activation is promoted by the action of the reticular formation that starts the general excitation. Amongst other things, the anterior cingulate cortex changes the priorities of attention and concentration (Botvinick et al., 2001), whilst the frontal cortex generates the plan of priorities for attention skills and working memory (Rushworth et al., 2004).

If not valued as stressful, the body’s response would involve the implementation of normal, specific homeostatic mechanisms that are appropriate for the particular situation. If it is considered stressful, the emergency mechanisms that constitute the stress response (activation of SMA and HPA axes, autonomic arousal and neuroendocrine activation) would become activated (Finsterwald and Alberini, 2014). This may occur because the stimulus is qualitatively stressful for all individuals of a particular species or because it exceeds a certain threshold of intensity.

When the nerve-center activation appears to have peaked, autonomic activation occurs, activating the SMA axis. This, in turn, prepares the body to cope with the external threat and to facilitate state of fight-or-flight response, so that the internal environment is maintained uniform (homeostasis; Finsterwald and Alberini, 2014).

Once the reticular formation has begun the process of general activation, the hypothalamus is excited. The hypothalamus is responsible for controlling the functions of the autonomic nervous system and the endocrine system, organizing survival behaviors such as fighting, feeding, fleeing and reproduction (Maggi et al., 2015). After the arrival of the information to the hypothalamus, the primary and quick response will cause the release of catecholamines (noradrenaline and adrenaline) from the hypothalamus and the sympathetic pathway. These catecholamines are responsible for putting the body on alert in preparation for “fight–or-flight”. Some of the observable physiological effects include tachycardia, increased blood pressure, sweating and dilated pupils (Ursin and Olff, 1993). Finally, the neuroendocrine response is activated, initiated by the HPA axis when neurons in the paraventricular nucleus (PVN) of the hypothalamus secrete a peptide called corticotropin releasing factor (CRH). This and other related hormones enter the private circulatory system connecting the hypothalamus with the anterior pituitary. Within seconds, CRH activates the pituitary by inducing the release of adrenocorticotropic hormone (ACTH; Leng and Russell, 1998). Once released, ACTH enters into the blood flow and stimulates the adrenal cortex to release GCs: cortisol, hydrocortisone and corticosterone. Mineralocorticoids, such as deoxycorticosterone and aldosterone, alongside sex steroids, such as progesterone, are also released. The release of GCs in stressful situations aims to raise the level of glucose in the blood, helps to convert fats into energy, increases blood flow, and stimulates behavioral responses; at the same time it inhibits unnecessary vegetative activities such as digestion, growth, reproduction and immune system (Stratakis and Chrousos, 1995), to prepare the body to deal with the emergency.

### Corticoids

Glucocorticoid receptors (GRs) are ligand dependent transcription factors that regulate the expression of several genes. The appropriate modulation of its expression is critical for maintenance of cellular homeostasis. Genomic actions of steroids on CNS are mediated by two types of receptors (de Kloet et al., 1987, 1993): the glucocorticoid receptor type II (GR) and the mineralocorticoid receptor type I (MR). Both differ from each other in their specificity and affinity for ligands, their location and their function. Inactivated GRs have a predominantly cytoplasmic localization. Free receptor forms a complex with a system of chaperone proteins (heat shock protein or hsp90, hsp70 and hsp40) or with FKBP52 (immunophilins; Rajapandi et al., 2000; Galigniana et al., 2001; Pratt et al., 2001). These proteins confer to the receptor high affinity for the hormone and dissociate from the receptor upon binding the hormone. However, these proteins also seem to be involved in the movement of GRs from the cytoplasm to the nucleus along microtubules (Galigniana et al., 2001; Pratt et al., 2001). When the hormone binds to the receptor, the complex undergoes a conformational change that mediates its activation and facilitates the binding of specific DNA sequences to regulatory or promoter regions of genes. GCs therefore control gene expression by activating or inhibiting specific genes.

MR primarily binds to GCs such as corticosterone and mineralocorticoids such as aldosterone, presenting high affinity for both, whereas GRs have relatively low affinity for corticosterone and higher affinity for cortisol and various synthetic GCs such as dexamethasone. MRs are present in target organs for mineralocorticoids such as kidney, intestine and salivary glands, where they regulate the osmotic balance and salt intake. At the central level, MRs are particularly expressed in the hippocampus, septum, amygdala, olfactory bulb and in regions of the cerebral cortex (de Kloet et al., 1993). Within the hippocampal formation, they abound in the CA1 and CA3 areas as well as in the dentate gyrus, and they have also been found in lesser amounts in the nucleus of the solitary tract. On the other hand, GRs present a more widespread distribution and are therefore present in all tissue types. However, within the CNS, they are more abundant in the limbic system (septum and hippocampus), in PVN and in brainstem monoaminergic areas.

The distinct affinity of both receptors for corticosterone determines a different degree of occupation depending on circulating levels of GCs. Thus, when the concentration of GCs is high, as occurs in rats during the night or after stressful situations, GRs are the receptors involved. In contrast, MRs are involved during the light phase, when they play a modulatory role in the basal levels of the HPA axis (de Kloet et al., 1987). At the peripheral level, almost all tissues possess GRs and, in a lesser extent, MRs (Munck et al., 1984). The varying distribution of these receptors in tissues allows GCs to act in different metabolic processes that are essential for body homeostasis. Finally, this general vegetative excitation returns to the brain, initiating a feedback on internal receptors in the body. This feedback may result in a further increase in the overall excitation (sympathetic stimulation) and, conversely, a relaxation (parasympathetic stimulation) by decreasing the excitation.

The effects of GCs on the stress response are important and necessary. Physiological changes associated with stress include mobilization of energy to maintain brain and muscle function, (which sharpens and focuses the attention of the perceived threat), increased cerebral perfusion rates and local cerebral glucose utilization, enhanced cardiovascular output and respiration, redistribution of the blood flow, and modulation of immune system (Carrasco and Van de Kar, 2003; Filipović and Pajović, 2009). However, its long-term activation can have harmful effects on health, such as increased blood pressure, muscle tissue damage, atherosclerosis, diabetes, inhibition of immunological responses (Sorrells and Sapolsky, 2007; Tank and Lee Wong, 2015), and even damage in brain structures such as the hippocampus (Sapolsky, 1986; McEwen, 1999).

### Models of Stress

Depending on the time of exposure, different stress models have been developed (Jaggi et al., 2011). Exposure to a stressor can last from seconds to days or weeks, and the effects of stress on the endocrine system depend on the duration of the exposure and its impact. If the stressful stimulation is repeated and prolonged in time, it is considered chronic stress and has been associated with important psychological and physiological conditions in humans.

Two types of chronic stress can be distinguished: continuous chronic stress and intermittent chronic stress. In chronic continuous stress, the animal may be successively subjected to stress for days or weeks, whereas in intermittent chronic stress animals are exposed for a period of weeks to a stressful situation, with daily exposure time ranging from minutes to hours. As an example of a model of continuous stress, we could mention social stress in rodents. This is particularly noticeable when the aggressive behavior of the male is enhanced by the presence of females (Blanchard et al., 1993).

The stress model most used experimentally is the intermittent chronic stress model, in which animals are exposed to a stressful situation on daily basis. In the literature, the most commonly used models are exposure to cold, restriction of movement (in a tube), forced swimming and electric foot shock among others (Katz et al., 1981; Armario et al., 1988; Riccio et al., 1991). The chronic variable stress is another model of chronic stress that shares characteristics with the two types of chronic models listed above; the animals are daily exposed to different random stressors, making it difficult to predict the arrival of a particular stimulus and avoiding the consequent adaptation (Katz et al., 1981; Armario et al., 1985, 1988).

## Dual Roles of Glucocorticoids in Microglia Activation and Polarization

From a historical perspective, GCs are strongly associated with anti-inflammatory hormones, as exemplified by the 1950 Nobel Prize in Physiology or Medicine, awarded jointly to Edward Calvin Kendall, Tadeus Reichstein and Philip Showalter Hench *“for their discoveries relating to the hormones of the adrenal cortex, their structure and biological effects”*. In keeping with this view, the action of GCs in the brain could be solely seen as inhibitors of pro-inflammatory microglia under conditions of brain inflammation. However, accumulating evidence from the peripheral and the central nervous system have revealed that, under specific conditions, GCs may promote brain inflammation including the pro-inflammatory activation of microglia. It is, however, important to highlight that GC actions do not necessarily have to fully mimic stress actions. From studies in the peripheral system, it has been established that the timing and duration of the stress response may be critical for delineating an immune response (Sorrells et al., 2009). If stress takes place before immune activation, it may sensitize (prime) immune cells for a greater response. In this section, we will briefly summarize literature demonstrating anti- and pro-inflammatory actions of GCs on brain. Special attention will be paid to the pro-inflammatory actions of either GCs or stress in the CNS, considering their relevance in the course of different neurodegenerative diseases.

### Pro- and Anti-Inflammatory Actions of GCs

As already mentioned, the timing of GC administration may be critical in finally determining a pro- or an anti-inflammatory action. Supporting this idea, it has been shown that *in vitro* treatment of microglia with GCs decreases the ability of these cells to produce interferon-gamma (IFN-γ) and tumor necrosis factor-alpha (TNF-α) in response to stimulation with lipopolysaccharide (LPS; Tanaka et al., 1997). In similar conditions, dexamethasone suppresses LPS-induced nuclear factor kappa B (NF-κB) activation in the brain (Glezer et al., 2003). Interestingly, NF-κB has been involved in the resolution of inflammation (Lawrence and Fong, 2010; Taoufik et al., 2011). Similarly, GCs suppress microglial toxic radical production (Colton and Chernyshev, 1996; Drew and Chavis, 2000) and cell proliferation (Woods et al., 1999). Exogenous corticosterone abolishes cytokine gene expression in microglia (Blais et al., 2002; Nadeau and Rivest, 2003). Together, these studies point out that GCs are able to directly inhibit microglia function and suggest a possible role for GCs in the regulation of microglia function *in vitro*. Stress-related GCs, when applied after an inflammatory challenge, are also anti-inflammatory. Thus, post-LPS acute stress decreases the production of pro-inflammatory cytokines (Goujon et al., 1995). Systemic LPS has been associated to GCs increase. Interestingly, adrenalectomy potentiates the increase of brain cytokines produced by systemic LPS. Consequently, GCs released upon LPS treatment inhibit the inflammatory response triggered by LPS.

Even though GCs may exert immunosuppressive actions on microglia, it is becoming evident that they may also play opposite roles, this effect being particularly evident if GCs or stress are applied before a pro-inflammatory challenge. The administration of GCs prior to a systemic LPS challenge exacerbates brain inflammation in terms of up-regulation of mRNA expression of interleukin-1β (IL-1β) and TNF-α in the hippocampus (Frank et al., 2010). However, under the same experimental conditions, GCs diminishes the expression of pro-inflammatory cytokines when given 1 h after LPS challenge (Frank et al., 2010). In this study, GCs act as pro-inflammatory agents at 2 and 24 h before LPS challenge, thus suggesting that the previous exposure to GCs could prime microglia (Frank et al., 2010). To demonstrate that microglia is the target of GC action, the authors isolated microglia from GC-pretreated rats and further challenged it *ex vivo* with LPS. Under these conditions, GCs treatment further upregulated MHCII and toll-like receptor-4 (TLR-4), and produced more IL-1β and TNF-α (Frank et al., 2010), supporting the hypothesis that GCs sensitizes brain microglia to subsequent pro-inflammatory stimuli. The pro-inflammatory actions of GCs may be neurotoxic under conditions associated to neural damage and inflammation. The effect of GCs under conditions of excitotoxicity induced by intrahippocampal kainic acid injections are associated to pyramidal CA3 degeneration and glial response (Dinkel et al., 2003). In this study, GCs increased the number of hippocampal immune cells including microglia, up-regulated mRNA and protein expression of IL-1β and TNF-α, and increased the kainate-induced lesions in the CA3 region (Frank et al., 2010). Further evidence that GCs exacerbate the microglial pro-inflammatory response under conditions of excitotoxicity derived from hippocampal cultures, where GCs exacerbates KA-induced expression of pro-inflammatory cytokines (Macpherson et al., 2005). Different stress paradigms, when applied before an inflammatory challenge, have also been shown to sensitize pro-inflammatory microglia. Thus, stress exposure to inescapable tail shock (Johnson et al., 2002, 2003, 2004) potentiated the expression of pro-inflammatory mediators (i.e., IL-1β, TNF-α and inducible nitric oxide synthase, iNOS) in hippocampus, hypothalamus and frontal cortex, as well as the sickness response (i.e., fever) produced by a peripheral injection of LPS given 24 h after the stressor regimen. Similar results were found after 14 daily sessions of unpredictable chronic stress (Munhoz et al., 2006) in terms of expression of pro-inflammatory mediators (TNF-α, IL-1β, iNOS). Subacute stress also induces GR-dependent microglia proliferation (Nair and Bonneau, 2006). In this study, the authors used a murine restraint stress model known to elevate GC levels. Stress maintained for 6 days increased microglia proliferation *in vivo*. This stress-induced proliferation of microglia is mediated by GCs since RU486 (or mifepristone), a potent inhibitor of GR activation, prevented this effect (Nair and Bonneau, 2006). Besides, N-methyl-d-aspartate (NMDA) receptor blockage prevented the proliferating stimulus induced by stress. Further work is needed to shed light into the role of NMDA receptors in microglia proliferation since NMDA receptor expression is mostly located on neurons. A paradigm of inescapable shock 24 h before microglia isolation from hippocampal tissue, and further plating and treatment with LPS, was used to test if microglia was the neuroimmune substrate responsible for the stress-induced pro-inflammatory response (Frank et al., 2007). This experimental design showed that stress primes microglia to a subsequent inflammatory challenge. Besides, it was found that stress downregulated CD200, a neuronal glycoprotein that maintains microglia in a quiescent state of activation (Hoek et al., 2000).

We and others have demonstrated that chronic stress potentiate the microglial pro-inflammatory response (de Pablos et al., 2006, 2014; Tynan et al., 2010; Espinosa-Oliva et al., 2011; Wohleb et al., 2011; Hinwood et al., 2012). Psychological chronic stress-induced inflammatory changes in microglia/macrophages were blocked by propranolol, an indication of an active role of β-adrenergic receptors in stress-induced microglial pro-inflammatory response (Wohleb et al., 2011). GRs but not MRs seem to have a central role in stress-induced sensitization of microglia. Blockage of GRs prevents stressor-induced enhancement of cytokine release (Munhoz et al., 2006). We and others have demonstrated that blockage of GRs prevents stress-induced microglial activation (Nair and Bonneau, 2006; de Pablos et al., 2006; Espinosa-Oliva et al., 2011). Similar results have been found using inhibitors of GCs synthesis (Nair and Bonneau, 2006). These studies suggest that GRs are key mediators in modulating the activation state of microglia.

**Figure 2:**
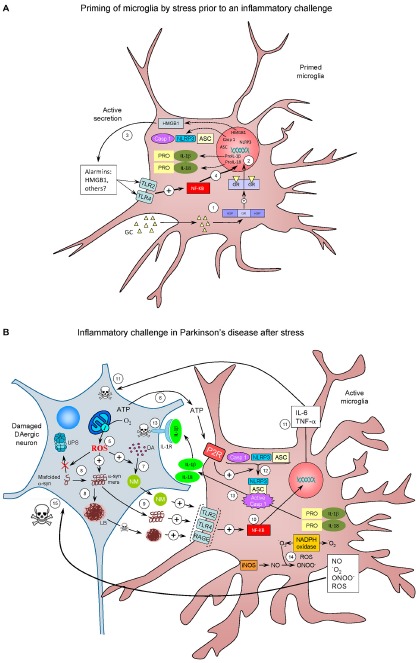
**The realm of vicious cycles. (A)** Microglia priming by stress prior to an inflammatory challenge. (1) Stress increases glucocorticoids (GCs) levels, which enter the microglial cell. Once inside, GCs can bind mineralocorticoid and Glucocorticoid receptors (GRs) or both (here referred to as GR). GR are normally bound to HSPs. Upon binding, HSPs are released from GR to further translocate to the nucleus and induce transcriptional machinery (2) that lead to active secretion of HMGB1 (3), an alarmin well-known for being an endogenous true-ligand of Toll-like receptors (TLR), including TLR2 TLR4. TLR activation is known to activate NF-κB (4), thus inducing transcription of the NLRP3 inflammasome machinery (NLRP3, ASC and caspase 1) along with expression of pro Il-1α and pro IL-18 proteins (caspase-1 substrates). Under these conditions, microglial cells become primed for a subsequent inflammatory challenge. **(B)** Inflammatory challenge in Parkinson’s disease (PD) after stress. Several factors (genetic and/or non genetic) may drive nigral DAergic neurons to an altered state in which ROS production (for example) are increased (5). Degenerating cells release different factors, including ATP (6), a well known inducer of NLRP3 assembly. ROS may induce an excessive production of the dark polymer neuromelanin (NM) (7), as well as impairs degradation of misfolded α-synuclein (α-syn) by the ubiquitin proteasome system (UPS) and potentiate the formation of α-syn oligomers (α-syn-mers) and Lewy bodies (LB), the most distinctive histopathological feature of PD (8). NM, α-syn oligomers and LB (this after neuronal death) are released from the dying DAergic neurons and recognized for different PRRs including TLR2, TLR4 and RAGE (9). Again, this leads to NF-κB activation and translocation to the nucleus (10), with the consequent transcription of pro-inflammatory genes (11); pro-inflammatory cytokines as TNF-α and IL-6 may induce neuronal death (11). ATP released from the damaged DAergic neurons activate the purinergic receptor (P2R) on microglial cells, leading to the assembly of the NLRP3 inflammasome and caspase-1 activation (12). Activated caspase 1 cleaves pro-Il-1α and pro-IL18 to IL-1β and IL-18, which are then released (13). IL-1β is known to bind IL-1R on DAergic neurons, which may contribute to cell death (13). Activation of microglia is accompanied by increased activity of different ROS- and RNS-producing enzymes such as iNOS, NADPH oxidase and myeloperoxidase (not shown) (14). NADPH oxidase catalyzes the production of superoxide anion (•O_2_) from oxygen in response to different pro-inflammatory stimuli. •O_2_ reacts with NO (mainly derived from upregulation of iNOS) to produce peroxynitrite (ONOO-), the most reactive free radical, thus inducing nitrosative stress. Peroxynitrite can both initiate and sustain a toxic loop eventually leading to neuronal damage (15), establishing a self-perpetuating process of neuroinflammation and neurodegeneration.

The role of IL-1β in the stress-induced sensitization of proinflammatory immune response and GC responses to a subsequent immune challenge has been analyzed (Johnson et al., 2004). Intracisternal administration of IL-1 receptor antagonist 1 h before tail shock completely prevented the stress-induced enhancement in central and pituitary IL-1β and plasma IL-6 release following LPS challenge (Johnson et al., 2004). Central human recombinant IL-1β administration significantly enhanced elevated central IL-1β levels and plasma corticosterone following LPS challenge. This study demonstrates a central role of IL-1β in mediating microglia brain priming to subsequent immune challenge. More efforts have been made to uncover how GCs sensitizes brain microglia to further pro-inflammatory stimuli, thus becoming neurotoxic. Within this context, and considering the central role of IL-1β in modulating GC-derived immune responses, the NLRP3 inflammasome (nucleotide-binding domain, leucine-rich repeat, pyrin domain containing proteins) appears as an attractive target where GCs may exert its pro-inflammatory action (Figure 2). NLRP3 senses various stimuli ranging from ATP, nigericin, uric acid crystals, *Escherichia coli* and *Citrobacter rodentium* (Gurung and Kanneganti, 2015). Activation of the NLRP3 inflammasome is tightly regulated and requires two independent signals: a priming step is first needed to drive transcription of inflammasome machinery. This first signal is usually facilitated through activation of TLRs or nucleotide-binding oligomerization-domain protein 2 (NOD2) to initiate the NF-κB signaling pathway. It should be first highlighted that GCs increase the activity of the transcription factor NF-κB (Hermoso et al., 2004; Smyth et al., 2004; Munhoz et al., 2006). Blockage of TLR2 and TLR4 prevents stress-induced priming of the microglial pro-inflammatory response, thus supporting the notion that pro-inflammatory actions of GCs relies, at least in part, on TLRs signaling (Weber et al., 2013). Once the machinery of NLRP3 inflammasome is synthesized due to the priming response, subsequent activation, referred to as “signal 2”, results in its oligomerization and inflammasome assembly, eventually leading to caspase-1-dependent cleavage and secretion of pro-IL-1β and pro-IL-18 (Figure 2). GC-induction of NLRP3 could serve as a mechanism for stress- and GC-induced priming of neuroinflammatory processes (Figure 2). GCs induce NLRP3 mRNAs and proteins in primary macrophages (Busillo et al., 2011). More interesting, these authors found that dexamethasone enhanced the ATP-induced release of IL-1β, an effect that was prevented by RU486, thus suggesting that GCs may sensitize macrophages-derived proinflammatory response by regulating NLRP3 expression and function (Busillo et al., 2011). This was confirmed by using a caspase-1 inhibitor, which completely inhibited the ATP-dependent and GC-enhanced release of IL-1β (Busillo et al., 2011). Subsequent studies suggested a similar effect of GCs on microglial cells (Frank et al., 2014). In this study, microglia was isolated from adrenalectomized rats and treated with different doses of GCs to be subsequently challenged with systemic LPS. A dose-response effect was found in terms of Iba-1, MHCII, NF-κBIα and NLRP3, in a concentration dependent manner. Activity of the NLRP3 inflammasome was not measured in this study. It would be interesting to test the effect of GC treatment on ATP or nigericin-induced NLRP3 assembly, caspase-1 activation and IL-1β release. Considering that ATP is released by injured or dying cells, these findings may explain why GCs enhances the immune response. Injured and dying cells release danger-associated molecular patterns (DAMPs). DAMPs and pathogen-associated molecular patterns (PAMPs) are detected by TLRs, thus activating the immune system (Figure 2).

Stress-induced release of DAMPs represents an attractive molecular link between stress and priming of brain microglia. Supporting this view, it has been demonstrated that inescapable tail shock induced high mobility group box-1 (HMGB-1), which is considered an archetypical alarmin (Frank et al., 2015) in the hippocampus, along with NLRP3 up-regulation (Weber et al., 2015). Intracisternal administration of BoxA, an HMGB-1 antagonist prior to stress, prevented the LPS-induced up-regulation of NLRP3 and proinflammatory cytokines in isolated cultured microglia (Weber et al., 2015). Interestingly, microglia isolated from animals subjected to inescapable tail stress was found to actively secrete HMGB-1, thus bringing a rationale of how acute/chronic stress or GCs may lead to release of one or different DAMPs, thus increasing the inflammatory response to a subsequent pro-inflammatory stimulus. Figure 2 highlights more potential mechanistic events linking chronic stress and PD.

## Effect of Chronic Stress in Animal Models of Parkinson’s Disease

Animal models of PD based on neurotoxins and the manipulation of genes implicated in this disease have been important in elucidating aspects as the molecular basis of dopaminergic neurons death or the importance of misfolded proteins in neurodegeneration, respectively (Dauer and Przedborski, 2003). Although research has deepened into the pathology and symptoms of PD, the factors involved in the onset and course of this disease remain to be characterized. Taken into account the pro-inflammatory effects of GCs on the CNS and the important role that inflammation plays in the development of PD, stress have been considered to influence not only the diversity in symptoms and course of PD within different patients, but also their individual responses to medication after the onset of the disease (Foley et al., 2004).

With regards to the effects of stress in different animal models of PD, recent studies have demonstrated that stress and high corticosterone levels can exacerbate nigral neuronal loss and motor symptoms in these animals (Smith et al., 2008; Kelly et al., 2012; de Pablos et al., 2014). This suggests that the impediment of functional and structural compensation generated by stress may be enhancing neurodegenerative processes and thus may represent a key factor in the pathogenesis of PD.

One of the first studies about the relationship between PD and stress showed that chronic stress and elevated corticosterone levels increased neurodegenerative events and motor deficits in a rat model of PD (Smith et al., 2008). It is generally acknowledged that intracranial injection of 4 mg/mL of 6-OHDA induces DA depletion (Kostrzewa and Jacobowitz, 1974; de Pablos et al., 2014). They evaluated the effects of both chronic restraint stress and oral treatment with corticosterone, a hormone released under stress conditions, on motor impairments and neurodegenerative processes in the unilateral rat model of PD. The authors showed that chronic psychological stress, such as that produced by daily restraint, and elevated corticosterone levels can separately lead to impaired skilled limb use in both naïve and 6-OHDA DA-depleted rats. Furthermore, in the 6-OHDA injected group, stress and corticosterone treatments also disturbed limb coordination and altered exploratory behavior, which was accompanied by an acceleration of the neurodegenerative processes and neuronal loss in the SN. All these findings point out that stress can increase functional deficits and thus accelerate the loss of DA-producing neurons (Smith et al., 2008).

Methamphetamine (METH) is known to cause damage to dopaminergic nerve terminals in the striatum, producing loss of TH and DA, and an activation of microglia and astroglia (Seiden et al., 1976; Hotchkiss et al., 1979; Bowyer et al., 1994; Miller and O’callaghan, 1994; O’callaghan and Miller, 1994; Ladenheim et al., 2000; Thomas et al., 2004; Fleckenstein et al., 2007; Krasnova and Cadet, 2009). To suppress this METH-related neuroinflammation, mice were pre-treated with corticosterone, both acutely (30 min before METH) and chronically (1 week before METH; Kelly et al., 2012). Not only both treatments failed to prevent neuroinflammation responses to METH, but the striatal neuroinflammatory response to METH was enhanced when mice were chronically pre-treated. This effect was further accompanied by enhanced astrogliosis and dopaminergic neurotoxicity. Furthermore, chronic pre-treatment also sensitized frontal cortex and hippocampus to induce a neuroinflammatory response to METH. These levels of chronic corticosterone are associated with high physiological stress, suggesting that chronic corticosterone therapy or sustained physiological stress may sensitize neuroinflammation and neurotoxicity responses produced by pro-inflammatory drugs as seen on METH (Kelly et al., 2012).

In another study showing the negative effects of stress/GCs (de Pablos et al., 2014), the authors observed that chronic stress increased both microglial activation and the expression of pro-inflammatory markers in an animal model of PD based on the intranigral injection of LPS. Importantly, they reported a higher inflammatory response in stressed animals which was associated with an increased death rate in dopaminergic neurons of the SN. To test whether this effect was related to GCs, they pretreated the animals with the GR inhibitor RU486. When the animals received this treatment, both microglial overactivation and the subsequent neuronal death in response to LPS were prevented, once again suggesting a potential negative role of stress/GCs in PD (de Pablos et al., 2014).

### Anti-Inflammatory Effects of GCs

However, besides their negative effects, GCs are the most powerful endogenous immunosuppressors via inhibiting the transcription of genes involved in the innate immune response, such as the pro-inflammatory transcription factor NF-κB (McKay and Cidlowski, 1999). Within this context, animals treated with RU486 showed a rapid and severe neurodegeneration after the intracerebral infusion of LPS (Nadeau and Rivest, 2003). They attributed this effect to impaired levels of cytokines in the cerebral environment, in particular TNF-α, as the inhibition of its biological activity was able to abolish the neurotoxic effect of LPS injection. According to these data, GCs could play a role as modulators of the innate immune system in the CNS; therefore, an imbalance on their immunosuppressive activities may lead to cerebral damage (Nadeau and Rivest, 2003).

As mentioned before, GCs exert their actions mostly through ubiquitously expressed type II GRs. Since several reports have hypothesized a link between GC-GR responses and PD pathogenesis, several studies have attempted to elucidate the role of these receptors in regulating dopaminergic neurodegeneration (Morale et al., 2004; Ros-Bernal et al., 2011). Ros-Bernal et al. (2011) performed 1-methyl-4-phenyl-1,2,3,6-tetrahydropyridine (MPTP) injections in mice in which GRs had been selectively knocked-out in either macrophages/microglia or dopaminergic neurons. MPTP treatment unleashed an increase in CNS GRs that was followed by a rapid increment in the number of microglia colocalizing these receptors. Mice lacking GRs in microglia but not in dopaminergic neurons experienced a higher loss of dopaminergic neurons after MPTP intoxication that could not be prevented by corticosterone. The increased death of dopaminergic neurons observed in the former was also correlated with reductions in DA uptake and DA levels in the striatum. Moreover, the absence of microglial GRs increased microglial reactivity and continuous activation. The regulatory role of GRs was confirmed by a higher expression of pro-inflammatory genes, such as TNF-α, alongside a decrease in anti-inflammatory genes, as IL1-R2. Together, these findings point out the potential role for the GC-GR system in the development of PD, and how GR dysfunction in PD may result in a subsequent chronic inflammatory reaction generating a positive feedback (Ros-Bernal et al., 2011).

Morale et al. (2004) injected MPTP in transgenic mice that constitutively expressed GR antisense RNA from early embryonic life. This produced a deficiency in GRs. They found this deficiency exacerbated the MPTP-induced toxicity to dopaminergic neurons, so that an increased loss of tyrosine hydroxylase positive nigral neurons and a decrease of DA levels in the striatum were observed. In these mice, microglia produced higher nitrite levels than in wild-type mice; these increases occurred before the loss of dopaminergic function. This shows the importance of GRs in the anti-inflammatory and immunosuppressive effects of GCs, and establishes a relation between impairment function of GRs and vulnerability to MPTP (Morale et al., 2004).

A question arising from these studies is how acute and chronic stress can trigger opposite effects in terms of brain inflammation and associated neurodegeneration. The paradoxical actions of GCs on microglial activation may be associated with differences in pharmacological approaches, experimental models or steroid hormone concentrations (Glezer et al., 2003; Macpherson et al., 2005). However, as mentioned earlier in this review, the timing and duration of stress response may be critical for modulating the immune response (Sorrells et al., 2009). Although evidence for a causal relationship between stress and PD has not been defined, stress could be playing an important role in its pathogenesis. GRs density differs throughout distinct brain structures and, interestingly, it is increased in regions involved in motor control, such as the motor cortex, basal ganglia and cerebellum (Ahima and Harlan, 1990; Ahima et al., 1992). This might in turn enhance their susceptibility to the effects of stress in both humans (Maki and Mcilroy, 1996) and rats (Metz et al., 2001, 2005).

Therefore, stress might represent a critical variable to be consider in the progression of neurodegenerative events underlying PD, where it can contribute to early onset of the motor symptoms in the presymptomatic stage of the disease as well as worsen the symptoms of PD once the disease has been diagnosed (Smith et al., 2008).

## Chronic Stress and Parkinson’s Disease in Humans

Within the medical community, it is generally accepted that chronic stress can be a contributing factor in the development of mental illness, metabolic diseases and immune system suppression, amongst many other physiological dysfunctions. For instance, it has been recently discovered a genetic relationship between anxiety and hypertension through microRNA regulation of the cholinergic pathway (Hanin et al., 2014). Higher risk of developing a neurological disease after prolonged emotional stress has already been established in AD (Rothman and Mattson, 2010). Although this relationship has not been established yet in PD, some studies prove that there is a higher prevalence of PD in people suffering stress. For instance, depression, as well as other chronic stress situations, increase the prevalence of PD (Schuurman et al., 2002) and seems to be related to severe symptoms like dyskinesias, disturbed sleep, and bradyphrenia (Pålhagen et al., 2008). The relationship between these two pathologies is not well-defined yet. Some studies estimate a 50% prevalence of depression in PD patients compared to 11% in non-PD patients (Hemmerle et al., 2012). Depression has also been proposed as a symptom for some subtypes of PD (Brown et al., 2011). However, this poorly understood relationship is due to an overlap of symptoms between both diseases. Defining depression as a previous stage, a concomitant disease, a non-motor symptom or an emotional stress factor is complicated because depression does not follow the same rhythm as other symptoms during PD progression. A possible explanation may be a deficit in neurotransmitters such as norepinephrine (Espay et al., 2014) or serotonin (Fox et al., 2009), suggesting a complementary field of action of chronic stress in PD through these neurotransmitters (Fitzgerald, 2014).

The idea of emotional stress being implicated in PD onset has already been supported in some PD patients who previously suffered the Holocaust (Salganik and Korczyn, 1990). Former American prisoners of war have roughly twice the rate of death due to PD (Page and Tanner, 2000). During World War I the term “shell shock” (currently known as post-traumatic stress disorder) was coined to describe those soldiers affected by a diversity of mental disorders resulting from combat stress (Crocq and Crocq, 2000). In most severe cases, many of the symptoms observed often remarkably resembled those of PD (Linden et al., 2013; Djamshidian and Lees, 2014). Among the motor symptoms recorded, they found mask-like expressions, resting tremors, postural instability, bradykinesia (slow movements), rigidity and freezing. Since intense traumatizing_events can trigger immediate “shell shock” symptoms resembling PD, it is reasonable to think that mild, but chronic stress may also elicit “shell shock”-like symptoms such as PD in the long run.

Chronic stress can induce pro-inflammatory cytokine and chemokine networks. These networks in turn cause a prolonged activation of the HPA axis (Haddad et al., 2002; McEwen, 2003) creating a cycle that exacerbates inflammation. TNF levels are increased during long-lasting stress periods in healthy volunteers (Visnovcova et al., 2015). Increased concentration of TNF has been found also in the SN of PD patients (Hirsch and Hunot, 2009). Further, it has been found a significant correlation between TNF levels and non-motor symptoms such as cognition, depression and disability in PD patients (Menza et al., 2010). On the other hand, regulatory T cells (CD4^+^ CD25^+^ FoxP3^+^) are responsible of maintaining self-tolerance and controlling immune responses. In this sense, it was found a 48% reduction in the proportion of regulatory T cells in post-traumatic stress disorder patients vs. controls (Sommershof et al., 2009). Similarly, a study reported lower CD4^+^/CD8^+^ ratios and a 24% reduction of CD4^+^ CD25^+^ cells (reaching up to a 64% reduction for CD4^+^ CD25^high^ cells) in PD patients (Baba et al., 2005). DA by itself is able to suppress regulatory T cells and activate resting effector T cells (CD4^+^ <<< CD8^+^), suggesting an important link between stress, lymphocytes and PD (Levite, 2015).

A review on the literature about the causes of PD concluded that emotional trauma could be a causative agent (Schwab and Zieper, 1965). In that article, the authors cited two specific cases. In one, an individual learned via telegram that his son had died in a plane that was shot down; in another, a woman saw her husband being killed in a car accident. In both cases, symptoms of PD emerged within hours, although such signs had never been previously detected. There is also a reported case of a woman who experienced sudden onset PD 1 week after discovering that her husband was involved in an affair (Zou et al., 2013). However, another study with more than 13,500 patients showed that the risk of PD was up to 42% lower among men that had experienced several major life events than those without any. No other correlation was found for women (Rod et al., 2010). The authors explained these results in part due to the fact that PD patients tend to be more cautious and have premorbid personalities, minimizing risk exposure, which could reflect an inherited tendency. In this sense, frontotemporal dementia and parkinsonism has been linked to chromosome 17 (Hong et al., 1998) and some forms of PD are inherited. They also argue that not all individuals respond equally to major life events.

Therefore, major life events do not necessarily reflect perceived stress by the individual and its allostatic load, meaning the wear and tear effect on the body (McEwen, 2007). Chronic stress develops from accumulation of little everyday life events and should, therefore, be approached differently in studies involving stress and PD. Some authors have established that stressors, such as job-related events, economic hardship, disappointment in love, loss of relatives and friends and social isolation, may be risk factors for developing PD due to the important role they play in the mental state of the individuals affected (Smith et al., 2002; Salmon, 2006; Hemmerle et al., 2012). The risk of parkinsonism in different occupations has been studied and authors found that those with daily exposure to dangerous and/or stressful situations are common occupations of people who suffer from PD (Goldman et al., 2005; Tanner et al., 2009). In keeping with this view, Indiana became in 2009 the first state in the U.S.A. to recognize PD as a line of duty disability among firemen, policemen and EMS responders. Moreover, according to another study with almost ten thousand participants, vital exhaustion (a psychological response when a person is unable to solve or adapt to the source of stress) may be a pre-motor marker of neurodegeneration leading to PD (Clark et al., 2013). A symptom of vital exhaustion is unusual fatigue. Interestingly, mental fatigue, a typical feature of patients with chronic fatigue syndrome, can be found in up to 70% of PD patients at some stage of the illness (Friedman et al., 2011). Chronic fatigue syndrome can increase some classical PD symptoms in depressed patients including slow thinking (bradyphrenia), psychomotor retardation or psychomotor agitation (Lane et al., 1991; Djamshidian and Lees, 2014). In fact, abnormalities in walk movement were detected in patients with chronic fatigue syndrome (Boda et al., 1995), suggesting a possible motor dysfunction similar to that seen in PD patients. Stress can increase the symptoms of PD, such as tremor after episodes of anxiety or anger (Djamshidian and Lees, 2014).

Although typical clinical features of PD include motor symptoms, the disease also include some non-motor disturbances, such as autonomic dysfunction as well as neuropsychiatric problems such as depression, mood changes and pain. It is known that stress can transiently increase motor symptoms of PD, and a positive association between cortisol and gait deficits has been shown (Charlett et al., 1998). Several studies also discuss how stress may deteriorate the symptoms of PD patients, such as bradykinesia and akinesia, the difficulty to initiate movements, sudden motor blocks (freezing), and tremor (Schwab and Zieper, 1965). Moreover, stress can also affect some nonmotor signs of PD. Individuals with PD have reduced hedonic responses after exposure to emotional stress (Macht et al., 2007). These authors demonstrated that hedonic responses of PD patients were reduced by stress, being this effect independent from depressive mood. The authors suggested that the ability to respond in an emotionally positive manner to external stimuli may be reduced in PD.

Stress has also been considered to influence not only the diversity in symptoms and course of PD within different patients, but also their individual responses to medication after the onset of the disease (Foley et al., 2004). This might depend on the extension of dopaminergic damage, so that stress could make abruptly appear an altered behavior that was hidden in a preclinical phase. Supporting this, experiments on rats showed that drugs enhancing dopaminergic function reversed neurological deficits induced by stress, while harmful effects of stress were increased by dopaminergic antagonists (Snyder et al., 1985).

However, the greatest risk factor for PD appears to be age, since the symptoms of PD emerge preferentially after the age of 65. In this regard, it is important to note that dysfunctions in the stress response develop during the aging process. Hence, as an organism ages, the response of the HPA axis to stress becomes hyperactive and less efficient to return to former homeostatic conditions, thus exposing brain cells to higher levels of GCs for longer periods of time (Stein-Behrens et al., 1992). Taking into account that another factor in the late onset of PD is the increased vulnerability of DA neurons to insults, the deregulation of the HPA axis might render cells in the aged brain more susceptible to degeneration in the face of subsequent stress.

High levels of cortisol have been found in PD patients (Hartmann et al., 1997; Charlett et al., 1998; Djamshidian and Lees, 2014). High levels of cortisol provoked by a dysfunctional HPA axis have been associated to dopaminergic cell loss and motor disability (Müller and Muhlack, 2007, 2008). However, a relative diurnal decrease of 22% in cortisol secretion in PD patients vs. controls has been reported (Hartmann et al., 1997). They postulated that a decreased expression of hippocampal MRs in PD patients may explain such reduction (Hartmann et al., 1997). All these data point out that stress could be an important factor in the development of PD. Measurement of stress hormones such as salivary cortisol levels (Djamshidian and Lees, 2014), inflammatory markers such as cytokines (Hirsch and Hunot, 2009), and the roles of physical exercise and cognitive behavioral therapy are potential lines of future research that may further clarify the role of stress in PD.

## Concluding Remarks

Accumulating evidence demonstrates how stress-related GCs may sensitize microglia to subsequent pro-inflammatory challenges, thus enhancing the brain inflammatory response (Figure 2). Excessive pro-inflammatory microglia activation may be neurotoxic, thus bringing a rationale between chronic stress and the progression of different neurodegenerative diseases, particularly PD. The potential involvement of brain inflammation in the etiology of PD is well established. Consequently, stress-related GCs may be an important contributing factor to modulate the long-term brain inflammatory response, including the appearance of neurotoxic microglia. Since midbrain dopaminergic neurons are especially sensitive to pro-inflammatory microglia, the impact of chronic stress in the etiology and course of PD deserves a special attention.

## Conflict of Interest Statement

The authors declare that the research was conducted in the absence of any commercial or financial relationships that could be construed as a potential conflict of interest.
